# Randomized crossover clinical trial of a Mindfulness-based intervention for nurse leaders: A pilot study

**DOI:** 10.1590/1518-8345.6548.4101

**Published:** 2024-03-15

**Authors:** Teresa Maria dos Santos, Alexandre Pazetto Balsanelli, Káren Mendes Jorge de Souza

**Affiliations:** ^1^ Universidade Federal de São Paulo, Escola Paulista de Enfermagem, São Paulo, SP, Brazil.; ^2^ Scholarship holder at the Coordenação de Aperfeiçoamento de Pessoal de Nível Superior (CAPES), Brazil.

**Keywords:** Mindfulness, Emotional Intelligence, Resilience, Psychological, Leadership, Nursing, Clinical Trial, Atención Plena, Inteligencia Emocional, Resiliencia Psicológica, Liderazgo, Enfermería, Ensayo Clínico, Atenção Plena, Inteligência Emocional, Resiliência Psicológica, Liderança, Enfermagem, Ensaio Clínico

## Abstract

**Objective:**

to analyze the effects of a Mindfulness-based intervention on nurse leaders’ emotional intelligence and resilience.

**Method:**

a pilot study of a randomized crossover clinical trial. The sample (n=32) was randomized into Group A (n=18) and Group B (n=14) and evaluated at the pre-test, post-test and follow-up moments. The outcomes were assessed using the Emotional Intelligence Measure, the Connor-Davidson Resilience Questionnaire and the Five Facet Mindfulness Questionnaire, and analyzed using Generalized Linear Mixed Models.

**Results:**

a total of 32 nurses with a mean age of 42.6 years old were evaluated. The analyses showed significant interactions between the effects of the group x moment factors on the Self-motivation (p=0.005), Sociability (p<0.001), Self-control (p=0.013), and Total (p=0.002) emotional intelligence skill scores; as well as on the Observe (p=0.042), Describe (p=0.008), Non-judgment (p<0.001), Act with awareness (p=0.004) and Total (p<0.001) mindfulness facets. Post-test: there was a statistically significant increase in the Sociability (p=0.009) and Self-control (p=0.015) emotional intelligence skills; as well as in the Non-judgment (p=0.022) and Total (p=0.002) mindfulness facets. Follow-up: a significant increase was observed in the Non-judgment (p=0.024) and Total (p=0.026) mindfulness facets. The “resilience” variable did not present statistical significance in the “group x moment” factor, both in the post-test and during follow-up.

**Conclusion:**

the Mindfulness-based intervention used proved to be effective in increasing nurse leaders’ emotional intelligence and dispositional mindfulness skills. Brazilian Registry of Clinical Trials (RBR-3c62gy), registered on March 4 ^th^ , 2020, updated on September 16 ^th^ , 2022.

## Resumen

**Objetivo:**

analizar los efectos de una intervención basada en *mindfulness* sobre la inteligencia emocional y la resiliencia de enfermeros líderes.

**Método:**

estudio piloto de un ensayo clínico aleatorizado cruzado. Muestra (n=32) aleatorizada en el grupo A (n=18) y grupo B (n=14), se los evaluó en el pretest, postest y seguimiento. Los resultados fueron evaluados mediante la Medida de Inteligencia Emocional, el Cuestionario de Resiliencia de Connor-Davidson y el Cuestionario *Mindfulness* de Cinco Facetas, y analizados por el *Generalized Linear Mixed Models* .

**Resultados:**

fueron evaluadas 32 enfermeras con una edad promedio de 42,6 años. Los análisis mostraron interacciones significativas de los efectos de los factores grupo vs. momento en los puntajes de las habilidades de automotivación (p=0,005), sociabilidad (p<0,001), autocontrol (p=0,013) y total (p=0,002) de inteligencia emocional; de las facetas observar (p=0,042), describir (p=0,008), no juzgar (p<0,001), actuar con conciencia (p=0,004) y total (p<0,001) de *mindfulness.* Postest: hubo un aumento estadísticamente significativo de las habilidades de sociabilidad (p=0,009) y autocontrol (p=0,015) de inteligencia emocional; de las facetas no juzgar (p=0,022) y total (p=0,002) de *mindfulness* . Seguimiento: se observó un aumento significativo en las facetas no juzgar (p=0,024) y total (p=0,026) de *mindfulness* . La variable resiliencia no presentó significación estadística en el factor grupo vs. momento en el postest y seguimiento.

**Conclusión:**

la intervención basada en *mindfulness* utilizada demostró ser eficaz para aumentar la inteligencia emocional y las habilidades de *mindfulness* disposicional de los enfermeros líderes. Registro Brasileño de Ensayos Clínicos (RBR-3c62gy), registrado el 4 de marzo de 2020, actualizado el 16 de septiembre de 2022.

## Resumo

**Objetivo:**

analisar os efeitos de uma intervenção baseada em *mindfulness* na inteligência emocional e resiliência de enfermeiros líderes.

**Método:**

estudo-piloto de ensaio clínico randomizado cruzado. Amostra (n=32) randomizada em grupo A (n=18) e grupo B (n=14), avaliados nos momentos pré-teste, pós-teste e seguimento. Os desfechos foram avaliados pela Medida de Inteligência Emocional, pelo Questionário de Resiliência Connor-Davidson e pelo Questionário das Cinco Facetas de *Mindfulness* , e analisados pelo *Generalized Linear Mixed Model* .

**Resultados:**

foram avaliadas 32 enfermeiras com idade média de 42,6 anos. As análises mostraram interações significativas dos efeitos dos fatores Grupo x Momento nos escores das habilidades de automotivação (p=0,005), sociabilidade (p<0,001), autocontrole (p=0,013) e total (p=0,002) de inteligência emocional; das facetas observar (p=0,042), descrever (p=0,008), não julgar (p<0,001), agir com consciência (p=0,004) e total (p<0,001) de *mindfulness* . Pós-teste: houve aumento estatisticamente significante das habilidades de sociabilidade (p=0,009) e autocontrole (p=0,015) de inteligência emocional; das facetas não julgar (p=0,022) e total (p=0,002) de *mindfulness* . Seguimento: observou-se aumento significativo das facetas não julgar (p=0,024) e total (p=0,026) de *mindfulness* . A variável resiliência não apresentou significância estatística no fator Grupo x Momento, no pós-teste e seguimento.

**Conclusão:**

a intervenção baseada em *mindfulness* utilizada mostrou-se efetiva no aumento das habilidades de inteligência emocional e *mindfulness* disposicional de enfermeiros líderes. Registro Brasileiro de Ensaios Clínicos (RBR-3c62gy), registrado em 04 de março de 2020, atualizado em 16 de setembro de 2022.

**Highlights:**

**(1)** Unpublished study of a Mindfulness-based intervention with nurse leaders.

**(2)** Positive effect on the participants’ emotional intelligence and mindfulness.

**(3)** Advances in knowledge about emotional intelligence and leadership resilience

**(4)** It encourages the implementation of sensitive and innovative health strategies.

## Introduction

 Leadership exerts an important impact on the nurses’ practice and the perception of a healthy working environment ^(^ ^1^ ^)^ . However, nursing managers’ life and work experience does not necessarily make them more effective at understanding, encouraging and supporting the team of professionals in adverse or emotionally challenging situations ^(^ ^2^ ^)^ . 

 A growing body of literature highlights the importance of resilience for nursing leaders ^(^ ^3^ ^-^ ^5^ ^)^ . Resilience refers to a process of active decision-making and conscious effort to move forward in an integrated and insightful way, following lessons learned from a conflicting experience ^(^ ^6^ ^)^ . Resilient leaders have effective coping patterns when solving problems in adverse situations and manage their feelings intelligently, influencing the well-being and creativity of the entire team ^(^ ^7^ ^)^ . 

 High resilience, emotional intelligence and mindfulness levels can equip nurse leaders to deal with adverse conditions, maintaining a sense of purpose, balance and well-being, with deeper reflection and resilience in open and trusting relationships, through motivation, feedback and self-knowledge ^(^ ^8^ ^-^ ^9^ ^)^ . Emotional intelligence can be defined as an individual’s ability to recognize and manage their own emotions and those of others, to guide their own thinking and regulate their behavior ^(^ ^10^ ^)^ . 

 Within a broader conceptual and practical ethical framework, Mindfulness derives from the Buddhist tradition. The empirical literature refers to the term as a mental state of consciousness that integrates attentional control of a chosen object and the way in which people pay attention to their experiences by identifying sensations, emotions and thoughts with curiosity, openness and acceptance. It involves training a certain consciousness quality that allows the practitioner to “respond” to emotionally stressful situations instead of “reacting automatically” to them ^(^ ^11^ ^)^ . During a working day, for example, managers can allow themselves to stop and practice mindfulness in order to reflect and regulate their emotions before making an important decision or in the face of a conflict between team members ^(^ ^12^ ^-^ ^13^ ^)^ . 

 Recent studies linking the mindfulness, emotional intelligence and resilience variables suggest that greater mindfulness skills help nursing professionals and students to promote compassionate care, personal well-being, resilience and emotional intelligence ^(^ ^14^ ^)^ , as well as in improving occupational burnout, with results in reducing errors and ensuring patient safety ^(^ ^15^ ^)^ . 

 Mindfulness-Based Interventions (MBIs), carried out during the COVID-19 pandemic, are known for their effectiveness in increasing mental health self-care, well-being and life satisfaction among nurses ^(^ ^16^ ^)^ . 

 The MBI benefits for nurses and nursing students are well documented in the literature in terms of reducing stress, anxiety, depression and burnout symptoms, as well as increasing empathy, well-being, and satisfaction with life and work ^(^ ^17^ ^-^ ^21^ ^)^ . Despite the value of this evidence, there is still little research exploring the effects of these interventions associated with the constructs of emotional intelligence and resilience for nursing leaders. The integration of mindfulness in the leadership context supports leaders in managing with greater awareness, self-reflection and recognition of the multiple perspectives and complexities of the system ^(^ ^22^ ^)^ . 

 The World Health Organization recommends that all health professionals be protected against the consequences of chronic stress and the development of mental health problems during the COVID-19 pandemic, including suggesting that leaders can model self-care strategies to mitigate stress ^(^ ^23^ ^)^ . Resilience and the cultivation of compassion in the workplace should be encouraged, so that caregivers can cope with crisis situations and deal with suffering and death ^(^ ^24^ ^)^ . 

The objective of this pilot study was to analyze the effects of a Mindfulness-based intervention on nurse leaders’ emotional intelligence and resilience. As a hypothesis, it was assumed that the intervention used, inspired by the Breathworks Mindfulness for Health (BMfH) program, would increase nurse leaders’ emotional intelligence and resilience as a primary outcome, and that there would also be an increase in the mindfulness levels among these professionals, as a secondary outcome.

## Method

### Type of study

Pilot study of a randomized crossover clinical trial, with pre-test, post-test and follow-up measurements, in a sample divided into two groups: Group A (n=18), and Group B (n=14).

 Pilot studies play an important role in improving the conduction and quality of a main randomized clinical trial, as they add the intention to elucidate uncertainties around feasibility of the method to be used and identify potential refinements and acceptability of the intervention for future field research, in terms of the causal mechanisms of change and optimizing its impact ^(^ ^25^ ^-^ ^26^ ^)^ . 

### Locus

 The study included nurse leaders from a large public university hospital located in the city of São Paulo, São Paulo, Brazil; and units of the Municipal Health Department belonging to the São Paulo City Hall ( *Secretaria Municipal de Saúde-Prefeitura Municipal de São Paulo* , SMS-PMSP), located in the city of São Paulo, SP, Brazil, which had Family Health Strategy Teams. The data were collected in the city of São Paulo, SP, Brazil. 

### Period

Dissemination of the research and recruitment and selection of candidates took place between January and March 2021, through electronic means and the Press Office media of the Federal University of São Paulo. The data were collected between April and September 2021 in the city of São Paulo, SP, Brazil.

### Selection criteria

Those included were graduate nurse leaders who worked in the selected hospital and integrated outpatient clinics, as well as in the SMS-PMSP Family Health Strategy Teams. Those who participated in any regular meditation practice were excluded, as well as those who reported problems with alcohol and drug abuse, and those who were undergoing treatment for any mental disorder diagnosis, in use of psychiatric medication.

### Definition of the sample

In this first stage, a pilot study was carried out with 35 randomized participants, 18 for Group A and 17 for Group B. After the pre-test, 3 Group B participants were lost, leaving 32 to be analyzed in the end.

A sample calculation was carried out for the main study, in which the emotional intelligence outcome variable was used, which inferred 166 participants, in order to have an 80% chance of finding a mean difference of 0.5 points between before and after and a standard deviation of 2.3 points, considering a 5% significance level. However, it was decided to carry out a pilot study in this research due to the COVID-19 pandemic prolongation period, which was responsible for restricting face-to-face activities in the place where dissemination, recruitment and data collection would be carried out.

### Participants and intervention

The volunteers who applied to take part in the study totaled 49 professionals. Of these, 35 met the inclusion criteria, answered the Informed Consent Form (ICF), agreed to take part in the study and were randomized and allocated, as follows: 18 participants in Group A and 17 in Group B. Randomization took place by simple draw, carried out by one of the study researchers. After the post-test, three Group B participants were lost, and 32 subjects were analyzed: 18 in Group A and 14 in Group B.

 The mindfulness intervention used in this study was inspired by the BMfH program ^(^ ^27^ ^)^ , adapted for online use as a result of the COVID-19 pandemic. This program consists of a psycho-educational structure, with informal (such as brushing your teeth with total awareness) and formal (body scanning, breath anchoring, conscious movements, compassionate acceptance, treasure of pleasures, open heart, loving kindness and compassion) mindfulness concepts and practices, always led by a qualified instructor. The participants were handed in audio materials for practicing the meditations and encouraged to practice daily, for a mean of fifteen (15) minutes, what they had practiced in the previous meeting. The main differences between the program offered in this study and BMfH were the shorter duration of the sessions (one and a half hours each) and the fact that it was carried out online. 

The instructor of the program in this study is certified by the Breathworks Mindfulness Foundation, with long-standing experience in meditation practices and in teaching the program. The meetings were videotaped with the participants’ permission, in order to ensure fidelity to the intervention protocol and the quality of the content delivered.

### Study variables

The primary outcome variables were resilience and emotional intelligence. The mindfulness facets were the secondary ones. All the variables were measured in relation to the central tendency and dispersion of the scores on the instruments used in the study, comparing Group A to Group B at the pre-test, post-test and follow-up moments.

### Instruments used to collect the information

The sociodemographic questionnaire allowed analyzing the variables of age, gender, schooling, belief, marital status, hiring institution and time as a leader. These variables were measured using descriptive statistics (mean, standard deviation or percentages, depending on the nature of each variable).

 One of the instruments used was the Connor-Davidson Resilience Scale-25 Brazil (CD-RISC-25) ^(^ ^28^ ^)^ , validated in Brazil ^(^ ^29^ ^)^ , which has an alpha coefficient of 0.93 and an intraclass correlation of 0.84. The 25 items are answered on an ordinal scale from zero (Not at all true) to four (Almost always true). The items encompass 5 factors: a) Personal competence; b) Trust in one’s own instincts; c) Tolerance of adversity; d) Positive acceptance of change; and e) Control. The score is based on the total sum of all the items and varies from 0 to 100, with higher scores showing greater resilience. This measure has been tested in the general population, as well as in clinical samples, and shows solid psychometric properties, with good internal consistency and “test-retest” reliability ^(^ ^30^ ^)^ . 

The second instrument was the Emotional Intelligence Measure (EIM), developed and validated in Brazil and based on Goleman’s model, which aims at measuring information processing in relation to emotions and feelings experienced or observed in social interactions.

The instrument measures 5 emotional intelligence skills, divided into 59 items: Self-awareness (10 items, α=0.78); Self-motivation (12 items, α=0.82); Self-control (10 items, α=0.84); Empathy (14 items, α=0.87); and Sociability (13 items, α=0.82); which should be evaluated by the respondent using an ordinal frequency scale: Never (1), A few times (2), Oftentimes (3) and Always (4).

 Cronbach’s alpha (α) refers to the study of the factorial structure of the questionnaire and indicates its reliability. The scores are obtained from the total sum of the answers scored by the respondent and can vary from a minimum of 59 points to a maximum of 236, indicating lower and higher emotional intelligence levels, respectively. It is also suggested to analyze the scores obtained in the skill subsets by adding up the values marked by the respondents on the answer scale for each item in a given factor and then dividing the sum by the number of items in the factor ^(^ ^31^ ^)^ . 

 Finally, the Five Facet Mindfulness Questionnaire (FFMQ) ^(^ ^32^ ^)^ , validated in Brazil ^(^ ^33^ ^)^ was used, which assesses dispositional mindfulness skills as a multifaceted construct. 

The Brazilian version consists of 39 items, which can be answered on a Likert-type self-assessment scale from 1 (Never or Rarely true) to 5 (Almost always or Always true). For this instrument, the term Mindfulness is conceptualized, in its dispositional quality, as a multifaceted construct, assessed in five facets: 1. Observe (noticing and being aware of events and experiences; α=0.76); 2. Describe (ability to name and describe the experiences observed; α=0.76); 3. Act with awareness (paying attention to activities in progress, rather than performing them mechanically in “autopilot” mode; α=0.79); 4. Non-judgment (not evaluating or using value judgments for thoughts and emotions; α=0.78); 5. Non-reactivity (ability to experience feelings and thoughts without reacting to or being influenced by them; α=0.68); or based on the total score (α=0.81), which indicates a general mindfulness skill. Although there is a total FFMQ score, it is recommended to analyze the facet scores separately, for which the minimum and maximum values are as follows: Observe: 7 and 35; Describe: 5 and 25; Act with awareness: 5 and 25; Non-judgment: 8 and 40; Non-reactivity: 8 and 40. The total score is obtained from the sum of the facet scores, with a minimum of 39 points and a maximum of 195, indicating the minimum and maximum mindfulness levels, respectively.

### Data collection

**Figure 1:**
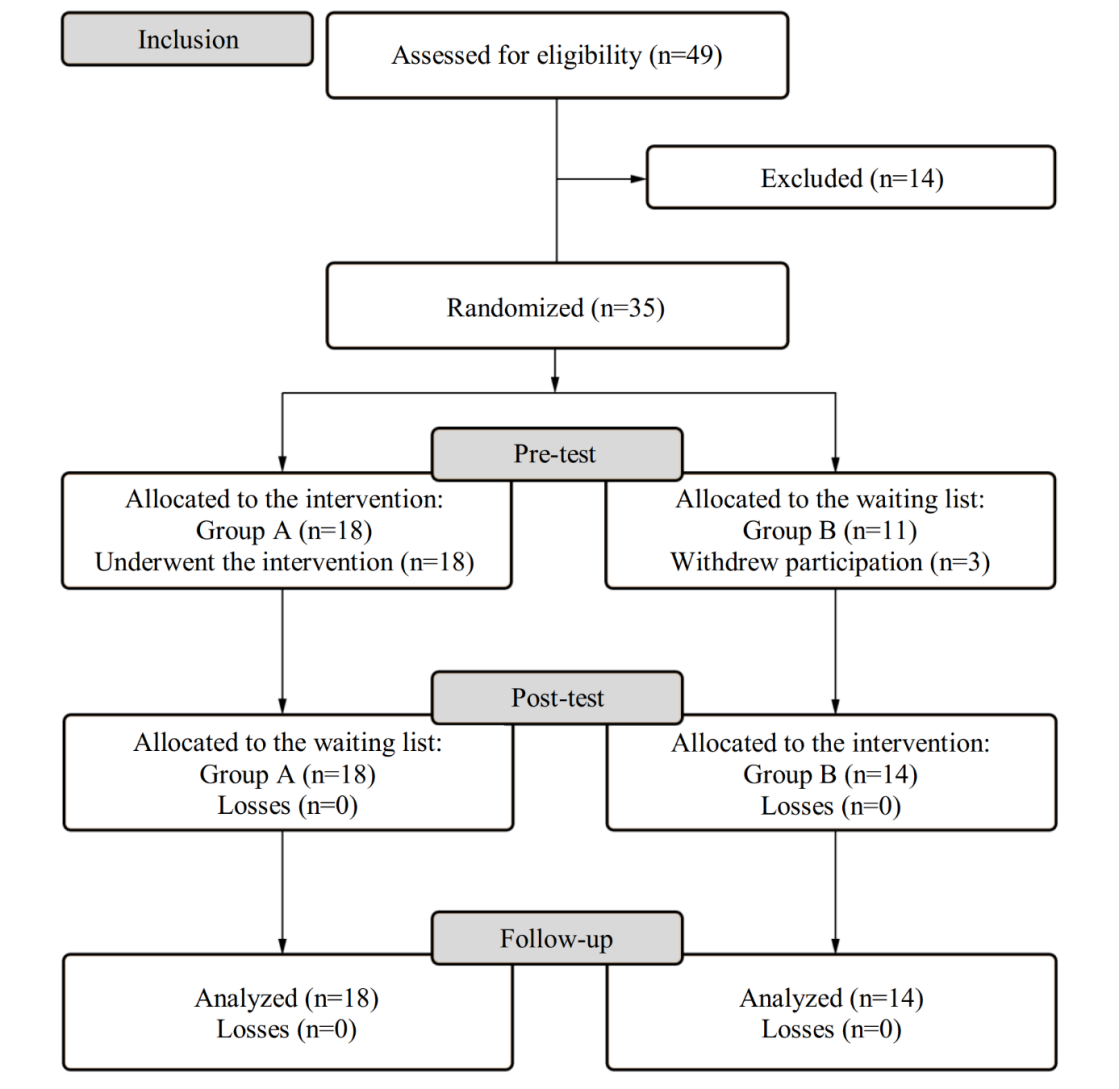
- Flowchart corresponding to the inclusion, allocation, follow-up and analysis of the participants, adapted from CONSORT. São Paulo, SP, Brazil, 2021

 Data collection took place between May and September 2021. After identifying the 49 eligible participants, 14 were excluded: 11 were undergoing psychiatric treatment (in use of medications); 2 practiced some form of meditation regularly; and 1 had a nursing degree but was employed as a nursing technician. The 35 participants who met the inclusion criteria were randomized and allocated to groups A and B. At the pre-test moment, the questionnaires were answered by all the participants (after which 3 from Group B withdrew their participation, leaving a sample of 32 participants). Subsequently, Group A took part in the intervention program between May and June 2021, whereas Group B remained on the waiting list. In July, both groups answered the questionnaires at the post-test moment. Once the questionnaires were completed and handed in, between August and September 2021, Group B took part in the intervention program (crossover), whereas Group A remained without any activity. At the follow-up moment in October 2021, all the participants filled in the questionnaires again. Figure 1 shows the flowchart for inclusion, allocation, follow-up and analysis of the study participants. 

 A two-month pause was taken between interventions in order to respect the wash-out period, which contributed to controlling a potential residual effect. Based on a previous study that applied wash-out periods of 32 hours for every 8 hours of mindfulness intervention ^(^ ^34^ ^)^ , it was assumed that the interventions in this study, which totaled 12 hours, would be carried out with a minimum wash-out period of 48 hours. 

### Data treatment and analysis

The data were analyzed using the International Business Machines - Statistical Package for the Social Sciences (IBM - SPSS) software, version 28.0, with a 5% (p≤0.05) statistical significance level for all tests.

Fisher’s Exact and Student’s t tests were used to compare the groups in relation to the sociodemographic variables. To measure the outcome variables, the groups were compared at the “pre-test” moment using Student’s t test for independent samples. The calculations for the Student’s t test were performed using the “bootstrap” sampling method with corrected and accelerated bias based on 1,000 samples. There was no violation of the homoscedasticity assumption (p>0.05, Levene’s test).

To check the effect of group x moment on the scores of the instruments used in the study, Generalized Linear Mixed Models (GLMMs) were developed. From the nature of the dependent variables and the testing of different adjustments based on the Akaike Information Criterion Corrected (AICC) and Bayesian Information Criterion (BIC), the best adjustment was obtained considering a gamma distribution with a log link function and a staggered identity covariance matrix for all the variables. Time and group were entered as fixed effects, whereas intersubject variability was entered as a random effect. The effects of the baseline measurements of each variable and the total scores of the other instruments on their baseline measurements were also controlled for, all of which were entered as fixed effects. All effects were calculated using robust estimation. Fit of these models was validated by visually inspecting the distribution of the standardized residuals using Q-Q plots.

 The effect size calculation, measured by Cohen’s d coefficient, was carried out to compare the groups, according to the moment and in relation to the estimated marginal means of the scores for the outcomes of interest. Cohen’s d coefficient interprets effect sizes as small between |0.200| and |0.499|; as medium between |0.500| and |0.799|; and as large when above |0.800| ^(^ ^35^ ^)^ . 

The CD-RISC evaluation took into account the instrument’s total value. In the case of EIM, the five emotional intelligence skills were analyzed separately (Self-awareness, Self-motivation, Self-control, Empathy and Sociability), in addition to the measure total score; and, as for FFMQ, the analyses considered the five facets (Observe, Describe, Act with awareness, Non-judgment and Non-reactivity) in isolation, as well as the total score obtained in the instrument.

### Ethical aspects

 The study was approved by the Research Ethics Committee belonging to the Federal University of São Paulo (proponent institution), approved in February 2021 under Opinion No. 4,524,698 and Certificate of Presentation for Ethical Appraisal ( *Certificado de Apresentação de Apreciação Ética* , CAAE): 20243419,5,0000,5505, as well as by the Research Ethics Committee of the São Paulo Municipal Health Department, co-participating institution, approved in March 2021 under Opinion No. 4,585,356 and CAAE: 20243419,5,3001,0086. 

 The ethical precepts were respected in accordance with Resolution 466/12 of the National Health Council ( *Conselho Nacional de Saúde* , CNS) ^(^ ^36^ ^)^ . The participants were fully aware of the intervention objectives and methods and were duly informed about secrecy and confidentiality of the data collected, by completing and accepting the Informed Consent Form (ICF). 

 The research was conducted in accordance with the study protocol, following the recommendations set forth in the Consolidated Standards of Reporting Trials (CONSORT), and registered in the Brazilian Registry of Clinical Trials (RBR-3c62gy), on March 4 ^th^ , 2020, and updated on September 16 ^th^ , 2022. 

## Results

As this is a pilot study protocol, the results are only expectable and are intended to assess feasibility and acceptability of the procedures defined for the intervention, estimate recruitment and retention of participants, define the sample size, and consider possible reformulations of the intervention used.

A total of 32 subjects (n=18 in Group A and n=14 in Group B) completed all the study phases and were analyzed. In relation to gender, 100% stated being female. Central tendency and dispersion measures for age according to group, as well as comparisons between groups, were carried out using Student’s t test for independent samples. The participants overall mean age was 42.6 ± 9.2 years old; Group A had a mean of 41.72 ± 7.99, and Group B recorded a mean of 42.71 ± 10.77. There was no statistically significant difference (p=0.770) when comparing the groups in relation to this variable.

Table 1 presents the descriptive analysis corresponding to the sociodemographic variables of the sample, comparing groups A and B using Fisher’s Exact test. The results show that there was no statistically significant difference (p>0.05) between the groups in relation to these variables, indicating that both groups are similar. 

**Table 1:** - Comparison of the scores obtained for the sociodemographic variables of the sample, with equivalence between Group A (n=18) and Group B (n=14). São Paulo, SP, Brazil, 2021

**Variable**	**Category**	**Group**						**p-value** ^*^
		**B**		**A**		**Total**		
		**n**	**%**	**n**	**%**	**n**	**%**	
Schooling	Higher Education	1	7,14	1	5,56	2	6,25	>0,999
	Specialization	11	78,57	14	77,78	25	78,13	
	MSc	2	14,29	3	16,67	5	15,63	
	PhD	0	0,00	0	0,00	0	0,00	
Belief	None	1	7,14	2	11,76	3	9,68	0,974
	Catholic	2	14,29	4	23,53	6	19,35	
	Spiritist	5	35,71	4	23,53	9	29,03	
	Evangelical	3	21,43	2	11,76	5	16,13	
	Afro-Brazilian	0	0,00	1	5,88	1	3,23	
	Eastern	0	0,00	0	0,00	0	0,00	
	Israelite	1	7,14	2	11,76	3	9,68	
	Others	2	14,29	2	11,76	4	12,90	
Marital status	Single	1	7,14	3	17,65	4	12,90	0,346
	Married	5	35,71	7	41,18	12	38,71	
	Divorced	4	28,57	3	17,65	7	22,58	
	Separated	1	7,14	1	5,88	2	6,45	
	Widowed	0	0,00	2	11,76	2	6,45	
	Stable union	3	21,43	0	0,00	3	9,68	
	Others	0	0,00	1	5,88	1	3,23	
Hiring institution	SMS	12	85,71	12	66,67	24	75,00	0,412
	HSP	2	14,29	6	33,33	8	25,00	
Time as leaders	Less than 1 year	3	21,43	0	0,00	3	9,38	0,326
	Between 1 and 3 years and 11 months	4	28,57	5	27,78	9	28,13	
	Between 4 and 6 years and 11 months	2	14,29	3	16,67	5	15,63	
	Between 6 and 9 years and 11 months	2	14,29	2	11,11	4	12,50	
	10+ years	3	21,43	8	44,44	11	34,38	

^*^ Statistically significant value at the 5% (p≤0.05) level

Table 2 presents the descriptive values and the comparative analysis of the groups in relation to the scores obtained in the CD-RISC, EIM and FFMQ instruments at the pre-test moment. The results showed that, at the pre-test (baseline), the groups presented significant differences only in relation to resilience (CD-RISC), indicating that they were similar in relation to the other variables, minimizing selection bias. 

**Table 2:** - Descriptive values and comparative analysis of the groups in relation to their scores on the CD-RISC*, EIM ^†^ and FFMQ ^‡^ , instruments at the pre-test moment. São Paulo, SP, Brazil, 2021

**Moment**	**Group**	**n**	**Mean**	**SD** ^§^	**p**	**ES** ^||^
CD-RISC *	B	14	99,64	12,06	0,029	0,798
	A	18	87,22	17,78		
EIM^† ^Self-motivation	B	14	37,43	4,64	0,420	0,276
	A	18	36,00	5,54		
EIM^† ^Sociability	B	14	34,50	3,65	0,729	0,125
	A	18	33,94	4,99		
EIM^† ^Empathy	B	14	38,57	5,40	0,584	0,182
	A	18	37,39	7,24		
EIM^† ^Self-awareness	B	14	27,86	3,48	0,248	0,389
	A	18	26,28	4,46		
EIM^† ^Self-control	B	14	29,00	5,19	0,482	0,265
	A	18	27,78	4,10		
EIM^† ^Total	B	14	167,36	13,18	0,294	0,354
	A	18	161,39	19,19		
FFMQ ^‡^ Observe	B	14	25,43	7,01	0,748	0,113
	A	18	24,67	6,56		
FFMQ ^‡^ Describe	B	14	29,29	5,14	0,203	0,440
	A	18	26,56	6,90		
FFMQ^‡ ^Non-judgment	B	14	24,21	6,76	0,940	0,027
	A	18	24,39	6,24		
FFMQ ^‡^ Non-reactivity	B	14	21,07	5,08	0,479	0,235
	A	18	19,67	6,57		
FFMQ ^‡^ Act with awareness	B	14	29,29	5,11	0,405	0,279
	A	18	27,61	6,60		
FFMQ ^‡^ Total	B	14	129,36	16,32	0,298	0,346
	A	18	122,89	20,29		

^*^ CD-RISC = Connor-Davidson Resilience Scale;

^†^ EIM = Emotional Intelligence Measure;

^‡^ FFMQ = Five Facet Mindfulness Questionnaire;

^§^ SD = Standard Deviation;

^||^ ES = Effect Size;

^¶^ Statistically significant value at the 5% (p≤0.05) level

Table 3 presents the GLMM investigation of the group x moment factor effects on each of the scores obtained in the study measures. All models were controlled for individual variability, each variable’s baseline measures and total baseline scores of the other instruments in the study. 

**Table 3:** - Investigation of the group x moment effect on the study measures. São Paulo, SP, Brazil, 2021

**Measure**	**Factor**	**Par.** *	**Effects**			
		**Degree of Freedom** **(DoF)**	**Intercept**	**Group**	**Moment**	**Group** x **Moment**
CD-RISC ^†^	--	DoF1,DoF2	6,31	1,28	1,30	1,30
		p	0,048 ^‡^	0,761	0,148	0,339
EIM ^§^	Self-motivation	DoF1,DoF2	6,33	1,27	1,30	1,30
		p	< 0,001 ^‡^	0,757	0,020 ^‡^	0,005 ^‡^
	Sociability	DoF1,DoF2	6,31	1,25	1,28	1,28
		p	< 0,001 ^‡^	0,206	< 0,001 ^‡^	< 0,001 ^‡^
	Empathy	DoF1,DoF2	6,30	1,27	1,30	1,30
		p	< 0,001 ^‡^	0,970	0,162	0,177
	Self-awareness	DoF1,DoF2	6,31	1,27	1,30	1,30
		P	0,008 ^‡^	0,888	0,748	0,930
	Self-control	DoF1,DoF2	6,31	1,27	1,30	1,30
		p	< 0,001 ^‡^	0,238	0,088	0,013 ^‡^
	Total	DoF1,DoF2	6,32	1,27	1,30	1,30
		p	< 0,001 ^‡^	0,587	0,002 ^‡^	0,002 ^‡^
FFMQ ^||^	Observe	DoF1,DoF2	6,30	1,27	1,29	1,29
		p	< 0,001 ^‡^	0,435	0,016 ^‡^	0,042 ^‡^
	Describe	DoF1,DoF2	6,33	1,27	1,29	1,29
		p	< 0,001 ^‡^	0,697	0,127	0,008 ^‡^
	Non-judgment	DoF1,DoF2	6,30	1,27	1,29	1,29
		p	0,001 ^‡^	0,965	0,314	< 0,001 ^‡^
	Non-reactivity	DoF1,DoF2	6,32	1,27	1,30	1,30
		p	0,139	0,920	0,248	0,150
	Act with awareness	DoF1,DoF2	6,31	1,27	1,30	1,30
		p	0,022 ^‡^	0,672	0,199	0,004 ^‡^
	Total	DoF1,DoF2	6,29	1,26	1,28	1,28
		p	< 0,001 ^‡^	0,564	0,025 ^‡^	< 0,001 ^‡^

^*^ Par. = Parameters;

^†^ CD-RISC = Connor-Davidson Resilience Scale;

^‡^ Statistically significant value at the 5% (p≤0.05) level;

^§^ EIM = Emotional Intelligence Measure;

^||^ FFMQ = Five Facet Mindfulness Questionnaire

This analysis showed significant interactions of the group x moment factor effects in relation to the scores of the Self-motivation (p=0.005), Sociability (p<0.001), Self-control (p=0.013) and Total (p=0.002) emotional intelligence skills, as well as in the scores of the Observe (p=0.042), Describe (p=0.008), Non-judgment (p<0.001), Act with awareness (p=0.004) and Total (p<0.001) mindfulness facets.

The CD-RISC scores in relation to the “empathy” and “self-awareness” emotional intelligence skills, as well as the Non-reactivity mindfulness facet, did not present statistically significant effects (p>0.05) in the interaction between the group and moment factors.

 Given that significant effects of the group x moment interaction were observed, Table 4 shall investigate the comparison analyses between the groups, according to the post-test and follow-up moments, in relation to the estimated marginal means of the scores. Student’s t tests with sequential Bonferroni correction for multiple comparisons were used for this analysis. The effect size was measured by calculating Cohen’s d coefficient. 

**Table 4:** - Comparison between the groups, according to moment, of the scores that presented statistical significance in Table 3 . São Paulo, SP, Brazil, 2021

**Variable** EIM* **FFMQ** ^†^	**Moment**	**Difference between the means** **(Group A -** **Group B)**	**Standard Error**	**95%** **Confidence** **Interval**		**p**	**Effect size**
				**Lower** **Limit**	**Upper** **Limit**		
EIM*-SM ^‡^	Post-test	1,40	1,53	-1,69	4,50	0,364	0,267
	Follow-up	-2,38	1,62	-5,67	0,90	0,150	0,495
EIM*- SOC ^§^	Post-test	3,30	1,19	0,88	5,73	0,009 ^||^	0,914
	Follow-up	-0,42	1,28	-3,02	2,19	0,748	0,111
EIM*-AC	Post-test	3,31	1,31	0,69	5,94	0,015 ^||^	0,803
	Follow-up	-0,73	1,40	-3,54	2,08	0,603	0,187
EIM*- Total	Post-test	8,86	4,42	-0,09	17,81	0,052	0,652
	Follow-up	-4,60	4,68	-14,07	4,87	0,332	0,323
FFMQ ^†^ - OBS**	Post-test	3,67	1,90	-0,14	7,48	0,059	0,541
	Follow-up	-1,36	2,18	-5,74	3,01	0,534	0,239
FFMQ ^†^ - DESC ^††^	Post-test	2,33	1,57	-0,85	5,51	0,146	0,470
	Follow-up	-1,22	1,66	-4,58	2,13	0,464	0,238
FFMQ ^†^ - NJ ^‡‡^	Post-test	6,17	2,59	0,95	11,38	0,022 ^||^	0,836
	Follow-up	-6,73	2,88	-12,53	-0,92	0,024 ^||^	1,153
FFMQ ^†^ - AWA ^§§^	Post-test	4,48	2,28	-0,10	9,07	0,055	0,680
	Follow-up	-2,93	2,48	-7,93	2,06	0,243	0,557
FFMQ ^†^ - Total	Post-test	17,85	5,33	7,14	28,56	0,002 ^||^	1,067
	Follow-up	-13,42	5,83	-25,14	-1,70	0,026 ^||^	0,852

^*^ EIM = Emotional Intelligence Measure;

^†^ FFMQ = Five Facet Mindfulness Questionnaire;

^‡^ SM = Self-motivation;

^§^ SOC = Sociability;

^||^ Statistically significant value at the 5% (p≤0.05) level ;

^¶^ SC = Self-control;

^**^ OBS = Observe;

^††^ DESC = Describe;

^‡‡^ NJ = Non-judgment;

^§§^ AWA = Act with awareness

 The results in Table 4 show that, at the post-test moment, there were statistically significant differences between the groups in the scores for the EIM-Sociability (p=0.009) and EIM-Self-control (p=0.015) skills; as well as in the scores for the FFMQ-Non-judgment (p=0.022) and FFMQ-Total (p=0.002) facets. These differences were accompanied by large effect sizes: EIM-Sociability [0.914]; EIM-Self-control [0.803]; FFMQ-Non-judgment [0.836]; and FFMQ-Total [1.067]. In all cases, Group A (which had finished the intervention) obtained a higher mean score when compared to Group B (which was on the waiting list). At the follow-up moment there were statistically significant differences between the groups in the scores for the FFMQ-Non-judgment (p=0.024) and FFMQ-Total [p=0.026] domains. The effect sizes in these cases were large both for FFMQ-Non-judgment [1.153] and for FFMQ-Total [0.852]. At the follow-up moment, Group B (which had undergone the intervention) started to present a higher mean score than Group A (which had received no intervention). 

For the other measurements, no statistically significant differences were observed between the groups, either at post-test or follow-up.

## Discussion

This study objective was to analyze the effects of a Mindfulness-based intervention on nurse leaders’ emotional intelligence and resilience. The results of this pilot study showed that, in relation to emotional intelligence, the analyses of the group x moment effect factors presented statistical significance in the scores of the Self-motivation, Sociability and Self-control domains and in the total emotional intelligence score. At the post-test moment, Group A had a higher mean score when compared to Group B, and a significant increase was observed in the emotional intelligence Sociability and Self-control domains.

 Similarly, recent studies have observed that mindfulness training was associated with increased emotional intelligence, contributing to the development of the practitioners’ ability to recognize their own emotions and those of others ^(^ ^37^ ^)^ , improve flexibility regarding cognitive re-evaluations of emotional information ^(^ ^38^ ^)^ , and increase self-perception and emotional regulation ^(^ ^39^ ^)^ . 

 The COVID-19 pandemic impacted the operation of health services and exacerbated the emotional strain on the teams, especially in relation to decision-making by leaders ^(^ ^40^ ^)^ . In line with other authors ^(^ ^41^ ^)^ , the increase in nurse leaders’ self-motivation, self-control and sociability in this study revealed their expertise in seeking stimuli within themselves to achieve the objectives proposed, in taking responsibility and managing healthy, ethical and true relationships, showing confidence despite the challenging and stressful circumstances. 

 Emotional intelligence is considered a critical aspect of leadership, and higher emotional intelligence levels have been associated with psychological well-being, better work performance by team members ^(^ ^42^ ^)^ and increased emotional balance and awareness, as well as with a reduction in the practitioners’ emotional exhaustion, this latter considered a key characteristic of the burnout syndrome ^(^ ^43^ ^)^ . 

 Although research studies on understanding leaders’ emotions are limited, mainly in relation to the interactions between leaders and employees ^(^ ^44^ ^)^ , the literature recognizes that lack of leadership and autonomy can contribute to nurse leaders’ emotional exhaustion, whereas recognition and rewards increase work-related well-being ^(^ ^45^ ^)^ . 

In relation to the mindfulness changes, the current study recorded significant alterations in the effects of the group x moment factors on the scores of the Observe, Describe, Non-judgment, Act with awareness and Total facets of dispositional mindfulness, as measured by FFMQ. After the intervention, there were significant changes in the Non-judgment facet and in the Total mindfulness score, with a large effect size. These results were maintained at the three-month follow-up, at which point Group B presented a higher mean score than Group A, showing the effect of the intervention after the crossover trial.

 In line with this evidence, other studies have revealed significant changes in scores in the Observe and Act with awareness domains of dispositional mindfulness, a significant increase in compassion, and a decrease in self-judgment and over-identification after practicing mindfulness ^(^ ^46^ ^-^ ^47^ ^)^ . 

 Mindfulness training can exert a force on the leader-employee relationship, promoting certain expansion of the employees’ emotional resources and well-being, as well as a reduction in emotional exhaustion and more tacit cooperation in the actions of the entire team ^(^ ^48^ ^)^ , with an influence on the employees’ performance, empowerment and job satisfaction ^(^ ^49^ ^)^ . 

 Other research studies into the effects of MBIs, carried out with nursing students, have revealed significant improvements in the mindfulness facets ^(^ ^50^ ^-^ ^51^ ^)^ . A piece of evidence designed to develop the emotional intelligence of professionals working in nursing homes also revealed the effectiveness of the mindfulness program in all facets, particularly in Observe, Act with awareness and Non-judgment ^(^ ^52^ ^)^ . When compared to medical leaders, a mixed-method study showed significant changes in mindfulness skills and ethical leadership competencies after the program ^(^ ^53^ ^)^ . 

 In the literature we have identified other results in relation to the mindfulness dimensions, although with differences in intervention styles, measures and populations. A randomized clinical trial carried out with nurses from a university hospital did not present statistical significance in the mindfulness levels after the intervention ^(^ ^54^ ^)^ . However, the instrument used in the aforementioned study only measures the attention/awareness facet and not all the facets, like FFMQ that we used. 

 Based on other scientific evidence ^(^ ^55^ ^)^ , we can argue that the nursing leaders in the current study have increased their ability to observe how their emotions affect their experiences, thoughts and behaviors; to verbally describe these experiences; to act with awareness, i.e., to pay attention to their activities in the present moment rather than behaving mechanically on “autopilot”; and to be non-judgmental about inner experiences, by allowing them to create distance and look in perspective. 

 In this study, the resilience variable was not statistically significant in the group x moment interaction, nor at the post-test or follow-up moments. Partial agreement with these findings can be seen in a paper on the effects of a Mindfulness program on Chinese hospital nurses. When comparing Group A to Group B, no significant group or time effect was identified on resilience, but the Group x Time interaction effect was statistically significant. There were also no significant changes in resilience post-intervention, only at the three-month follow-up. The practitioners allowed themselves to observe and describe negative affections or thoughts, resources that made them more adept at dealing with stress in a healthier and more adaptive way, in line with the literature ^(^ ^56^ ^)^ . 

 We found divergent results in the recent literature, in two previous studies where the participants reported increased resilience after a mindfulness intervention. One was carried out with employees of a company ^(^ ^39^ ^)^ and the other with military leaders ^(^ ^57^ ^)^ . In the latter, the positive changes in resilience observed by the interviewees were more frequently related to personal awareness of stress, evaluation of the effects of their behaviors and greater ability to lead others. However, it should be borne in mind that the resilience scales used in both studies are different from the one we used. 

 A research study into the impact of conscious leadership on the resilience and turnover intention of subordinate nurses dealing with COVID-19 patients revealed that conscious managers did not contribute to improving staff resilience, but did contribute to the turnover intention in times of crisis ^(^ ^58^ ^)^ . On the other hand, in this study we worked with leaders who participated in mindfulness-based practices to strengthen their own resilience. 

 There is a chance that resilience did not change significantly in this study because nurse managers had already built up a higher resilience level due to coping with the COVID-19 pandemic. A systematic review concluded that health professionals reported having moderate to high psychological resilience levels, despite the threat caused by the unknown virus and its consequences for mental health ^(^ ^59^ ^)^ . Resilience is an important protective factor for nurses ^(^ ^60^ ^)^ , although few research studies have been found on the influence of controllable work-related variables on nurses’ resilience, and the factors that contribute to high or low resilience levels are still unclear ^(^ ^61^ ^)^ . 

Among its strengths, the current study is the first to investigate the effects of an MBI inspired by the BMfH program, adapted to the online format, on resilience and emotional intelligence in nurse leaders. The significant increase in emotional intelligence and dispositional mindfulness, both in the group x moment interaction and at the post-test and follow-up moments, is strong evidence that the intervention produced significant effects on these variables, as the groups were only different when one of them had not yet undergone the intervention, and became similar after both groups had undergone the same intervention. In addition to that, there was stability in the gain obtained by the intervention in these variables, given that even after three months without the intervention, Group A remained equivalent to Group B when the latter had just undergone the intervention.

It should be noted that there were no sample losses in the post-intervention and follow-up, although data collection took place in the second year of the COVID-19 pandemic, with the imperative of social distancing, at a time when nurses were working overloaded, exhausted and mentally affected by the death of colleagues and family members, without time or motivation to carry out extracurricular activities. This fact points to the benefits of the program, which were recognized by the participants, which also contributed to minimizing the attrition bias. The participants’ interest in staying during the 8 weeks of the intervention points out to the academic community the importance of the IBM conducted as a relevant support for nursing leaders to act in a context of risk to mental and emotional health, such as the one experienced during the pandemic.

Discussions about the mindfulness practice included self-care and conscious communication by professionals with their work teams and organizations, gains that the professionals can carry with them for a long time.

 The pandemic has encouraged countless changes to traditional teaching and research modalities, especially the use of online interventions such as apps and video conferences, with success in improving the users’ compassion and mental health ^(^ ^62^ ^-^ ^63^ ^)^ . The current study contributed to the scientific support of an innovative technology in mindfulness training, consistent with periods of pandemics and other crises, similar to other findings in the literature ^(^ ^64^ ^-^ ^65^ ^)^ . 

 Although pilot studies have inherent limitations due to their small sample size, they represent a subset that reproduces the planned methodological path to be followed later. A pilot study has the potential to influence the decision to proceed to a subsequent main randomized crossover clinical trial; it makes it possible to evaluate recruitment, the protocol, the instruments used for data collection and analysis, the impact of the implementation strategy and the sample estimate, as well as the duration, efficiency and acceptability of the intervention ^(^ ^66^ ^)^ . 

Another limitation was the simple randomization used in this study, as it was carried out with professionals from Family Health teams who worked in health units linked to the Municipal Health Department, as well as with those who worked in hospitals and outpatient clinics. The organizational differences between the services involved can influence the nurse leaders’ performance and the results of the intervention tested. It is therefore suggested that, in the main study, randomization should take into account stratification of the nurses according to their workplace, guaranteeing homogeneity of nurses in the control and intervention groups.

We suggest that further studies, including the main one planned by the researchers in this research, with larger and more representative samples of the population, might help clarify these points and make the results more robust.

In addition to that, the program was only evaluated by means of a quantitative method, using self-report questionnaires. Possibly, a study with a qualitative approach included might provide elements for a more in-depth data analysis.

Future research, with more robust samples, might combine qualitative and quantitative approaches to explore the effects of MBIs among managers in greater depth and breadth.

The complexity of the leaders’ work in the health sector requires quick decisions, insight, creativity, interpersonal skills and maturity. The empirical and systematized evidence of this study can contribute to advancing knowledge by fostering discussions on the implementation of mindfulness programs as a powerful device for nurse leaders and organizations.

Sensitive approaches such as mindfulness training reveal great potential to assist nurse leaders in self-care, self-knowledge, self-compassion and genuine emotional intelligence for full leadership, both in times of crisis and in the everyday practice.

## Conclusion

From the results of this pilot study, the mindfulness-based intervention applied among nursing leaders proved to be effective in increasing these professionals’ emotional intelligence and mindfulness, and might influence the decision to carry out a main randomized crossover clinical trial.

The contributions of this study to nurse leaders’ work were to enable them to improve their ability to regulate attention and emotions; to improve their flexibility in cognitively re-evaluating information; and to develop skills such as self-motivation, self-control, interpretability, receptivity and self-assessment, which represent enormous help in overcoming crisis situations and stressful circumstances, as well as having the potential to contribute to healthier relationships between peers, patients and the organization. The increase in the leaders’ self-awareness made it possible for them to have deeper understanding of their personal limitations and greater ability to provide safe care, in accordance with professional standards, ethical stance and qualifications.

It is suggested that health organizations include mindfulness programs for nursing managers and teams, as simple and low-cost strategies, with the subtlety of including greater awareness and healthy management of emotions in the work environment, conferring greater sensitivity to competencies and decisions. This study can be used as a basis for future research on the benefits of MBIs for nursing leaders, considering the important transformations that an unprecedented crisis such as the COVID-19 pandemic has imposed on these professionals.
